# The Epidemiology and Clinical Management of Short Bowel Syndrome and Chronic Intestinal Failure in Crohn’s Disease in Italy: An IG-IBD Survey

**DOI:** 10.3390/nu16193311

**Published:** 2024-09-30

**Authors:** Tommaso Pessarelli, Matilde Topa, Andrea Sorge, Nicoletta Nandi, Daniela Pugliese, Fabio Salvatore Macaluso, Ambrogio Orlando, Simone Saibeni, Andrea Costantino, Francesco Stalla, Valentina Zadro, Lucia Scaramella, Maurizio Vecchi, Flavio Caprioli, Luca Elli

**Affiliations:** 1 Department of Pathophysiology and Transplantation, Università degli Studi di Milano, 20122 Milan, Italylucelli@yahoo.com (L.E.); 2 OU Internal Medicine and Gastroenterology, Fondazione Policlinico Universitario A. Gemelli IRCCS, 00168 Rome, Italy; 3 IBD Unit, “Villa Sofia-Cervello” Hospital, Viale Strasburgo 233, 90146 Palermo, Italy; 4 Gastroenterology Unit, Rho Hospital, ASST Rhodense, 20017 Rho, Italy; 5 Gastroenterology and Endoscopy Unit, Foundation IRCCS Ca’ Granda Ospedale Maggiore Policlinico, 20122 Milan, Italy; 6 Department of Medical Sciences, University of Turin, 10124 Turin, Italy; 7 Gastroenterology and Digestive Endoscopy Unit, Azienda USL Modena, 41012 Carpi, Italy

**Keywords:** short bowel syndrome in Crohn’s disease, prevalence of short bowel in Crohn, SBS in CD, intestinal failure in Crohn’s disease

## Abstract

Background/Objectives: Limited data exist on the epidemiology and clinical management of short bowel syndrome (SBS) and chronic intestinal failure (CIF) in Crohn’s disease (CD). This study aimed to evaluate these aspects in Italy. Methods: Members of the Italian Group for the Study of Inflammatory Bowel Disease (IG-IBD) were invited to complete a cross-sectional web survey. A subgroup analysis examined the influence of different clinical settings on SBS and CIF management in CD. Results: A total of 47/128 (36.7%) IG-IBD centers participated. Among them, 31.9% were teduglutide (TED) prescribers, and 48.9% were academic centers. The median estimated prevalence of CIF among small bowel CD patients was 1%, and it was significantly higher in academic centers (2.0% [IQR 1–5%] vs. 0.13% [IQR 0–1%], *p* = 0.02). Seventy-eight percent of centers managed fewer than 10 SBS and CD patients. Routine small bowel measurement and nutritional assessment were performed in only 15% and 42.6% of centers, respectively. TED was prescribed by 12 centers to 35 patients, with a treatment success rate exceeding 50% in 81.8% of centers. Conclusions: The estimated prevalence of CIF in CD patients with small bowel involvement in Italy is 1%. The diagnosis and management practices for SBS and CIF are suboptimal, and TED use is limited.

## 1. Introduction

Short bowel syndrome (SBS) and chronic intestinal failure (CIF) are rare, life-threatening conditions with poorly characterized epidemiology and management worldwide. SBS is defined as a small bowel length in continuity of less than 200 cm (measured from the ligament of Treitz) associated with clinical features of malabsorption [1]. SBS is recognized as the most common pathophysiological mechanism underlying CIF [1], which is defined as “a reduction of gut function below the minimum necessary for the absorption of macronutrients and/or water and electrolytes, such that intravenous supplementation is required to maintain health and/or growth”. According to this definition, CIF may result from either SBS or functional impairment of the small bowel, even when the small bowel length is more than 200 cm, a condition known as functional SBS [2].

Precise epidemiological data regarding the prevalence of both CIF and SBS are uncertain, as they are often derived from parenteral nutrition (PN) prescription registries [3]. Patients with small bowel Crohn’s disease (CD) frequently undergo surgery to treat stenoses, fistulas, and abscesses, although the need for surgery has decreased with the increased use and availability of biological therapies [3]. Data indicate that CD is the most common cause of CIF, representing 22.4–30% of cases, although a decline has been reported over the last few decades [3,4]. Longitudinal data from Japan show that 8.5% to 18.2% of CD patients develop CIF within the first 20 years of diagnosis [5,6].

The management of SBS and CIF requires a multidisciplinary team, including gastroenterologists, surgeons, radiologists, nutritionists, psychotherapists, and home-care nurses [7]. An accurate diagnosis of SBS requires direct (preferably surgical) measurement of the residual bowel. The mainstay of treatment for SBS associated with CIF is PN, which, however, is associated with a significant risk of complications and reduced quality of life [8]. Since the approval of the glucagon-like peptide-2 (GLP-2) analog teduglutide (TED), the medical management of patients with SBS and CIF has significantly changed. In carefully selected patients, such as those with higher baseline PN volume requirements, an absence of distal/terminal ileum or ileocecal valve (SBS type 1 and 2), and a lower percentage of remaining colon, TED has shown significant benefits in reducing intravenous fluid requirements, with a good safety profile [9,10,11]. However, real-life studies assessing epidemiological trends in TED prescription and its clinical efficacy in SBS and CIF, especially in patients with CD, are lacking.

This study aimed to analyze the epidemiology and clinical management of SBS and CIF among CD patients in Italy.

## 2. Materials and Methods

### 2.1. Survey Structure

We developed a questionnaire consisting of 22 multiple-choice or open-answer questions (Supplementary Materials Table S1) concerning the prevalence and clinical management of SBS and CIF among CD patients in various clinical settings in Italy. The questionnaire was revised and endorsed by the Scientific Committee of the Italian Group for the Study of Inflammatory Bowel Disease (IG-IBD). The first four items of the questionnaire pertained to the characteristics of the center, including the type of center (i.e., every center affiliated with a university and a medical specialization school was considered an academic center), its geographic location, the authorization to prescribe TED, and the prevalence of patients with small bowel CD (defined as L1, L3, or L4 according to the Montreal classification [12]) in each center. Eleven additional items assessed the epidemiology and clinical management of patients with CD and SBS, with a special focus on nutritional assessment. The last seven questions addressed the efficacy and adverse events of TED.

### 2.2. Survey Distribution

The invitation to participate in the survey was sent via email to all IG-IBD members on 26 July 2023. Participants could respond through an online application form at https://uk.surveymonkey.com (accessed on 26 July 2023). Two reminders were sent 30 and 60 days after the initial invitation. Data were anonymously collected on 31 October 2023. The study adhered to the Consensus for Reporting of Survey Studies (CROSS) guidelines for proper execution and reporting [13].

### 2.3. Statistical Analysis

The data were extracted into an anonymous Excel^®^ datasheet, and a statistical analysis was performed using IBM SPSS Statistics (release 24; IBM, Chicago, IL, USA). For categorical variables, expressed as absolute frequency numbers and relative percentages, we used Fisher’s exact test or χ^2^, as appropriate. If necessary, multiple-choice answers were grouped into dichotomous variables (e.g., dividing into >50% and <50% groups). For continuous variables, we calculated the mean with the confidence interval in cases of normal distribution, or the median and interquartile range in cases of non-normal distribution. We compared continuous variables using the Student’s *t*-test for normal distribution and the Mann–Whitney U-test for non-normal distribution. Confidence intervals were calculated at 95%, and *p*-values less than 0.05 were considered statistically significant. To assess whether the management of patients with SBS was impacted by the clinical setting and the expertise of the center, we performed subgroup analyses based on the clinical practice setting (i.e., academic or non-academic), the authorization to prescribe teduglutide, and the volume of CD patients (i.e., high-volume centers with >400 patients; low-volume centers with <400 patients).

## 3. Results

### 3.1. Participants

The questionnaire was sent to 683 IG-IBD members, representing 128 IBD centers. Seven responses (13.0%) were excluded due to incomplete questionnaires. Ultimately, 47/128 centers (37.3%) provided valid and complete responses to the survey.

**Figure 1:**
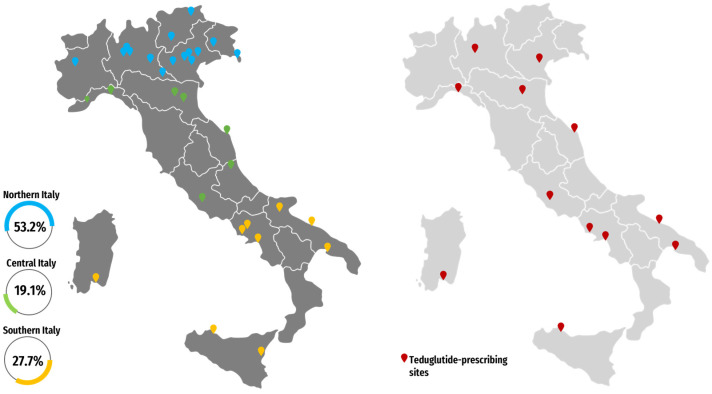
On the left, the geographical distribution of the 47 study participant centers; on the right, in red are identified the teduglutide-prescribing sites.

The participating centers were distributed throughout Italy (Figure 1), with a higher representation of northern Italian centers (53.2% [25/47]) compared to those from central and southern Italy (19.1% [9/47] and 27.7% [13/47]). About half of the centers (23/47, 48.9%) were academic centers. High-volume centers, defined as those following more than 400 patients with CD and small bowel involvement, accounted for 40.4% (19/47) of the total. Fifteen centers (31.9%) were authorized TED prescribers, distributed as follows: four (26.7%) from northern Italy, five (33.3%) from central Italy, and six (40%) from southern Italy (Figure 1). The characteristics of the responding centers are shown in Table 1.

### 3.2. Epidemiology

**Figure 2:**
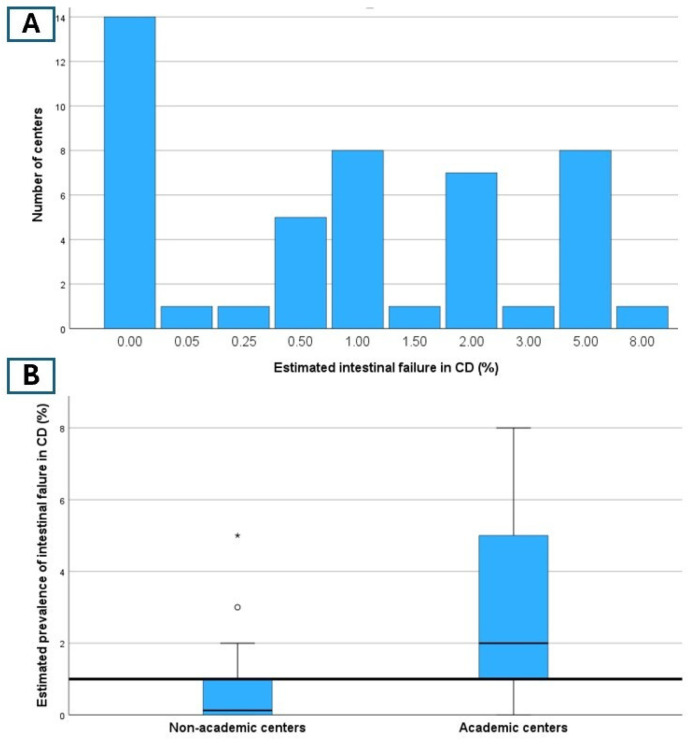
Estimated prevalence of intestinal failure in CD with small bowel involvement in all centers (panel **A**) and in academic vs. non-academic centers (panel **B**). ° and *: two outliers among non-academic centers.

The median overall estimated prevalence of intestinal failure in CD with small bowel involvement was 1% (IQR 0–2%, see Supplementary Materials Table S2 and Figure S1). A subgroup analysis revealed a significantly higher estimated prevalence of intestinal failure in academic centers compared to non-academic centers (2.0% [IQR 1–5%] vs. 0.13% [IQR 0–1%], *p* = 0.02) (Figure 2). A similar trend was observed in high-volume CD centers, although it did not reach statistical significance (0.5% [IQR 0–2%] vs. 1.5% [IQR 0.75–5%], *p* = 0.056).

**Figure 3:**
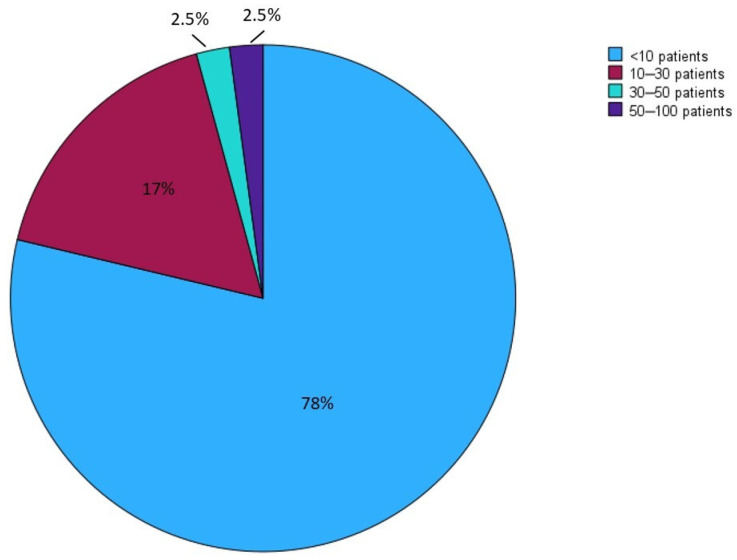
A cake graphic in which the responding centers are divided into four groups, according to the number of patients with SBS and CD with small bowel involvement reported to be followed in each center. The graphic outlines how most of the centers reported to follow less than 10 patients with SBS and CD, underlining the scanty centralization of patients affected by these conditions.

For the majority (63.9%) of CD patients with CIF, PN was necessary for less than four days per week. Thirty-five centers (74.5%) reported following fewer than 10 patients with SBS and CD, with only two centers (4.2%) reporting a case history of more than 30 patients (Figure 3). No statistically significant difference was found in the distribution of SBS patients between academic/non-academic centers, TED prescribers/non-prescribers, and high-volume/low-volume centers for CD.

SBS type 1 (terminal jejunostomy) [14] was the least frequent type of SBS reported, with 89.4% of centers reporting it in less than 25% of all CD patients with SBS.

### 3.3. Diagnostic Assessment

Direct surgical measurement of the residual small bowel length after surgery was routinely performed by 10.6% of the responding centers, with most centers (68.1%) performing it in less than half of Crohn’s patients undergoing small bowel surgical resections. Direct radiological measurement of the residual small bowel length after surgery was performed routinely in only five centers, two of which also regularly performed surgical measurements. A subgroup analysis did not reveal any statistically significant differences in the performance of these measurements between academic vs. non-academic centers, high-volume vs. low-volume CD centers, and TED prescribers vs. non-prescribers (see Supplementary Materials Table S2).

**Figure 4:**
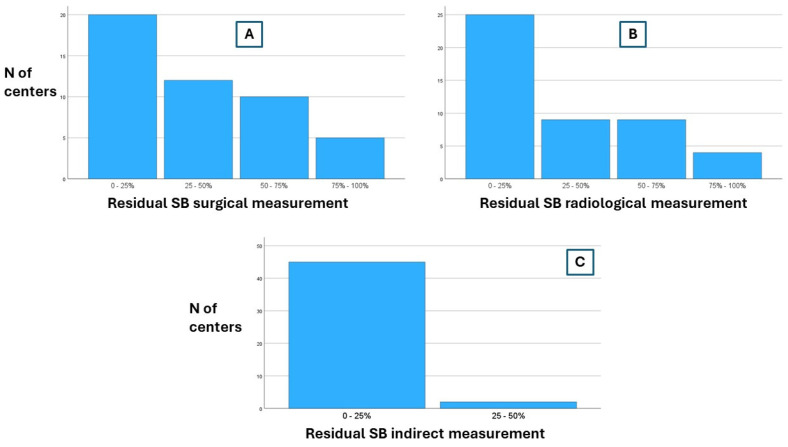
Percentage of patients undergoing direct surgical (panel **A**), radiological (panel **B**), or indirect (panel **C**) residual small bowel measurement in SBS and CD. SB: small bowel.

In their diagnostic work-up of CD patients with CIF, 95.7% of the responding centers reported not routinely using indirect tests (such as citrullinemia or D-xylose absorption tests) for residual bowel measurement (see Figure 4, Table 2).

### 3.4. Clinical Management

**Figure 5:**
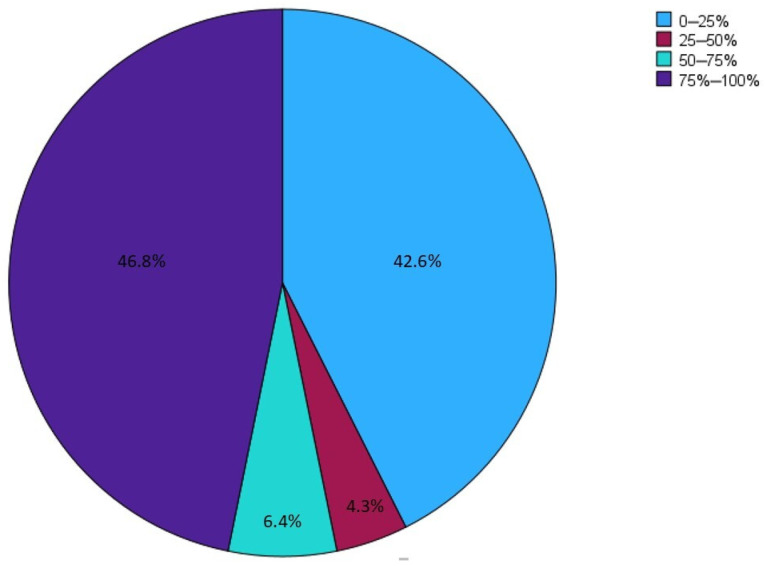
Nutritional referral in CD with small bowel involvement and CIF: overall data.

In 42.6% (20/47) of the responding centers, patients with CIF and CD are not routinely referred for nutritional evaluation and support (Figure 5). Academic centers referred patients for nutritional evaluation more frequently than non-academic centers (73.9% vs. 33.3%, OR 4.67, *p* = 0.008). The assessment of the nutritional status of CD patients with CIF was primarily (29 cases, 61.7%) based on clinical and biochemical parameters alone. In 27.7% of centers, radiologic nutritional markers (such as psoas muscle or paravertebral muscle measurement) were also used, while instrumental tools like bioelectrical impedance analysis were adopted in only 4.2% (2/47) of centers (Supplementary Materials Table S2). Symptomatic, non-disease-modifying pharmacological therapy (e.g., proton pump inhibitors, anti-peristaltic drugs, synthetic pancreatic enzymes, bile acid sequestrants) was regularly prescribed by 16.7% of the responding centers (Supplementary Materials Table S2).

### 3.5. Teduglutide

Among the responding centers, 31.9% (15/47) were TED-prescribing institutions. However, only 12 (80%) of them reported currently having patients on treatment. Overall, 35 patients with CD and CIF were reported to be currently treated with TED. The success rate of TED, defined as at least a 30% reduction in PN requirements [15], exceeded 50% of treated patients in 81.8% of the responding centers.

The maximum duration of TED therapy was less than 24 months in 63.3% of cases. Sixty percent of prescribing centers reported a reduction in the Crohn’s disease activity index (CDAI) after starting therapy with TED. Potential carcinogenic risk was a deterrent to TED prescription in 24% of respondents [16]. Among the centers where the drug is currently prescribed, three total TED-related adverse events necessitating the discontinuation of treatment were reported. All data regarding TED-related questions and responses are illustrated in Table 3 and in the Supplementary Materials Table S3.

## 4. Discussion

Our study estimates the prevalence of short bowel syndrome (SBS) and chronic intestinal failure (CIF) to be around 1% among inflammatory bowel disease centers in Italy, with a higher percentage observed in academic centers. However, the management of these conditions is often suboptimal, characterized by inadequate nutritional assessments and a limited access to TED prescriptions.

SBS and CIF are rare, life-threatening conditions that present significant management challenges and costs [17,18], requiring collaboration among multiple specialists [19]. CD is the primary cause of CIF [3] and one of the leading causes of SBS [2]. The introduction of TED therapy has positively influenced the clinical outcomes for many patients with SBS related to CIF and CD, as demonstrated in both clinical trials [10,11] and real-world studies [15,20,21,22,23,24,25,26]. Additionally, recent data indicate that TED therapy offers acceptable cost-effectiveness [27].

However, the scarcity and low reliability of available data on the epidemiology and management of SBS and CIF in CD patients [8] complicate the definition of the overall impact of these diseases and might obscure potential management deficits. To our knowledge, this is the first study attempting to address this knowledge gap, focusing on the epidemiology and management of SBS and CIF in CD patients in Italy.

We have made several noteworthy observations. First, the median estimated prevalence of CIF in CD with small bowel involvement in Italy is 1%. This result appears markedly inferior to the few available world data in the literature [5,6]. The suboptimal rate of residual bowel measurement in operated CD patients highlighted in our study suggests a potential underestimation of SBS prevalence in Italy, likely due to an inadequate diagnostic capacity.

Another important finding is the suboptimal management of patients with SBS, CIF, and CD in Italy. Measuring the residual bowel length in operated patients is crucial and should ideally be performed directly during the surgical procedure for an accurate diagnosis [2]. In Italy, this practice is routinely performed in only about 10% of centers, which may be due to inadequate communication between clinicians and surgeons or a lack of knowledge about the condition. Furthermore, there are no published data on the frequency of this practice in other countries. Indirect bowel measurements based on absorption markers are rarely utilized in Italy, likely due to the limited availability of these markers and their unsatisfactory diagnostic performance [28].

The multidisciplinary approach required in patients with SBS and/or CIF, including referral to an intestinal rehabilitation center with surgeons, gastroenterologists, radiologists, nurse coordinators, psychologists, and dieticians/nutritionists, is another key aspect of management. Our survey reported a very low nutritional referral rate, with 43% of centers not routinely referring CD patients with CIF associated with SBS for nutritional support. Academic centers reported performing this practice more frequently than non-academic centers, possibly due to the lack of clinical experience with SBS and CIF among IBD experts or the unavailability of clinical nutrition experts in non-academic settings.

Our study also highlighted TED prescription practices in Italy. The total reported number of patients currently on TED therapy was 35, which is relatively low, especially considering that TED-prescribing centers reported a median prevalence of intestinal failure in Crohn’s disease patients of 2%, with 60% of centers managing over 400 patients each. This remains the case even when accounting for the careful patient selection required for TED therapy. Potential reasons for this include a limited number of TED prescribers in Italy, the high costs of the therapy, and the inadequate centralization of these patients. Additionally, concerns about the theoretical carcinogenic effects of TED [29] may contribute to the low prescription rates. Despite these challenges, the reported success rate of TED (defined as a reduction of at least 30% in home PN requirements) was promising, with 81.8% of centers noting an improvement in over 50% of treated patients. This aligns with the existing literature [15,30], which also indicates few reported side effects necessitating drug suspension.

Our study has several strengths, including its originality, given the rarity of these conditions and the extremely limited available evidence. The potential direct clinical implications of our findings are significant. Our results raise several questions that future studies need to address. The validation of the survey questions by a group of “super-experts” in inflammatory bowel disease (IBD), along with the requirement for a single response per center and the homogeneous geographical distribution of respondents across Italian regions, enhances the reliability and validity of our study.

However, this study has limitations, including a suboptimal response rate, which aligns with findings from other IG-IBD surveys [31]. As is typical in surveys, the epidemiological data obtained consist of numerical ranges and estimated percentages, which limits our ability to provide absolute numerical data for large patient cohorts. Additionally, there is also a risk of response bias, considering the highly qualified cohort of respondents, many of whom were working in academic or TED-prescribing centers. However, the rarity of these diseases requires highly specialized management from tertiary referral centers, thus selecting responses from centers with high expertise. Nevertheless, to address this limitation and evaluate current epidemiological trends and clinical practices, we conducted subgroup analyses to examine differences in practice patterns based on IBD expertise and practice settings.

## 5. Conclusions

In conclusion, our study found an approximate 1% prevalence of CIF in CD patients in Italy. The current practices for diagnosing and managing SBS and CIF in Crohn’s disease are suboptimal, with the low rate of direct residual bowel measurement potentially leading to the underdiagnosis of these conditions. Furthermore, TED is prescribed in only a limited number of cases. These findings underscore the need for enhanced training in the management of these conditions, necessitating centralized care in high-volume, tertiary referral IBD centers equipped with intestinal rehabilitation services. Confirming these results with cross-sectional epidemiological studies would support prompt interventions to improve clinical practices and healthcare policies for managing these conditions in Italy.

## Figures and Tables

**Table 1:** Characteristics and expertise of participant centers.

Centers Included in the Analysis (*n*)	47
Academic centers, *n* (%)	23 (48.9)
Geographic distribution, *n* (%)	
Northern Italy	25 (53.2)
Central Italy	9 (19.1)
Southern Italy	13 (27.7)
Teduglutide prescribers, *n* (%)	15 (31.9)
Northern Italy	4 (26.7)
Central Italy	5 (33.3)
Southern Italy	6 (40.0)
Patient volume/center: Crohn’s disease with small bowel involvement, *n* (%)	
Less than 50	8 (17.0)
Between 50 and 200	16 (34.0)
Between 200 and 400	4 (8.5)
More than 400	19 (40.4)
Proportion of Crohn’s disease patients who underwent ≥1 intestinal resection/center, *n* (%)	
Between 0 and 5%	6 (12.8)
Between 5 and 15%	17 (36.2)
Between 15 and 30%	13 (27.7)
More than 30%	11 (23.4)

**Table 2:** Direct or indirect residual small bowel measurement in SBS and CD. Data from respondents and subgroup analysis.

Question	Total *n* = 47	Academic *n* = 23	Non-Academic *n* = 24	*p*	TED *n* = 15	Non-TED *n* = 32	*p*	>400 CD with SB Involvement *n* = 19	<400 CD with SB Involvement *n* = 28	*p*
Surgical measurement				0.088 *			1.000 *			0.751 *
0–25%	20 (42.6)	6 (26.1)	14 (58.3)		5 (33.3)	15 (46.9)		8 (42.1)	12 (42.9)	
25–50%	12 (25.5)	7 (30.4)	5 (20.8)		5 (33.3)	7 (21.9)		4 (21.0)	8 (28.6)	
50–75%	10 (21.3)	7 (30.4)	3 (12.5)		4 (26.7)	6 (18.8)		5 (26.3)	5 (17.9)	
75–100%	5 (10.6)	3 (13.0)	2 (8.3)		1 (6.7)	4 (12.5)		2 (10.5)	3 (10.7)	
Radiological measurement				0.290 *			1.000 *			1.000 *
0–25%	25 (53.2)	10 (43.5)	15 (65.2)		10 (66.7)	15 (46.9)		10 (52.6)	15 (53.6)	
25–50%	9 (19.1)	5 (21.7)	4 (16.7)		1 (6.7)	8 (25.0)		4 (21.0)	5 (18.9)	
50–75%	9 (19.1)	6 (26.1)	3 (12.5)		4 (26.7)	5 (15.6)		3 (15.8)	6 (21.4)	
75–100%	4 (8.5)	2 (8.7)	2 (8.3)		0 (0)	4 (12.5)		2 (10.5)	2 (7.1)	
Indirect measurement (citrullinemia, D-xylose absorption test)										
0–25%	45 (95.7)			NA			NA			NA
25–50%	2 (4.3)									
50–75%	0 (0)									
75–100%	0 (0)									

* Calculated with Fisher’s exact test by dividing into 2 groups (>50% and <50%). TED: teduglutide; CD: Crohn’s disease; SB: small bowel; NA: not applicable.

**Table 3:** Data regarding TED prescription among responding centers.

Question	*n* = Respondents to the Single Question (%)
Maximal duration of TED therapy in SBS-IF and CD patients?	*n* = 11
- <6 months	0 (0)
- 6–12 months	4 (36.4)
- 12–24 months	3 (27.3)
- >24 months	4 (36.4)
Success rate (% of patients with at least a 30% reduction in the need for weekly parenteral nutrition)?	*n* = 11
- 0–25%	2 (18.2)
- 25–50%	0 (0)
- 50–75%	6 (54.5)
- 75–100%	3 (27.3)
Reduction in the CDAI since the introduction of TED?	*n* = 11
- 0–25%	4 (40.0)
- 25–50%	3 (30.0)
- 50–75%	2 (20.0)
- 75–100%	1 (10.0)
TED excluded due to cancer risk?	*n* = 26
- 0–25%	20 (76.9)
- 25–50%	3 (11.5)
- 50–75%	2 (7.7)
- 75–100%	1 (3.8)

TED: teduglutide; SBS-IF: patients with short bowel syndrome and intestinal failure; CD: Crohn’s disease; CDAI: Crohn’s disease activity index.

## Data Availability

The original contributions presented in this study are included in the article/Supplementary Materials; further inquiries can be directed to the corresponding author.

## Supplementary Materials

The following supporting information can be downloaded at: https://www.mdpi.com/article/10.3390/nu16193311/s1, Table S1: Characteristics of survey respondents—original version; Table S2: Questions related to epidemiology and clinical management and relative subgroup analysis—original version; Table S3: Questions related to teduglutide therapy—original version. Figure S1—The estimated prevalence (% on the *y*-axis) of intestinal failure in Crohn’s Disease (CD) with small bowel involvement, as reported by each of the 47 responding centers (*x*-axis).
