# Supplementary material for: The Epidemiology and Clinical Management of Short Bowel Syndrome and Chronic Intestinal Failure in Crohn’s Disease in Italy: An IG-IBD Survey
Source: Nutrients. 2024 Sep 30;16(19):3311. doi: 10.3390/nu16193311 (PMC11478841; doi:10.3390/nu16193311)
Supplement: Supplementary file 1 [file nutrients-16-03311-s001.zip › nutrients-3214143-supplementary.pdf]

**Caratteristiche demografiche**	**N=47 (%)**
Lavora in un centro accademico?	23 (48.9)
Qual è il nome del centro in cui lavora?	Risposta variabile
Distribuzione geografica*<br><br>- Nord<br>- Centro<br>- Sud	 <br>25 (53.2)<br>9 (19.1)<br>13 (27.7)
Il centro in cui lavora è riconosciuto come prescrittore diretto di Teduglutide?	15 (31.9)
Distribuzione geografica centri prescrittori teduglutide<br><br>- Nord<br>- Centro<br>- Sud	 <br>4 (26.7)<br>5 (33.3)<br>6 (40.0)
Quanti pazienti con diagnosi di malattia di Crohn con coinvolgimento dell'intestino tenue (L1, L3 o L4 sec classificazione di Montreal) sono in cura presso il Suo Centro?<br><br>- Meno di 50<br>- Tra 50 e 200<br>- Tra 200 e 400<br>- Più di 400	 <br><br>8 (17.0)<br>16 (34.0)<br>4 (8.5)<br>19 (40.4)
Quanti di questi pazienti hanno subito uno o più interventi chirurgici di resezione intestinale?<br><br>- Tra lo 0 ed il 5%<br>- Tra il 5 ed il 15%<br>- Tra il 15 ed il 30%<br>- Piu del 30%	 <br>6 (12.8)<br>17 (36.2)<br>13 (27.7)<br>11 (23.4)

Table S1 – Characteristics of the survey respondents – original version

***indirect data**

**Demographic characteristics**	**N=47 (%)**
Do you work in an academic center?	23 (48.9)
What is the name of the center where you work?	Variable response
Geographic distribution*<br>- North<br>- Center<br>- South	 25 (53.2)<br>9 (19.1)<br>13 (27.7)
Is the center where you work recognized as a direct prescriber of Teduglutide?	15 (31.9)
Geographical distribution teduglutide prescribing centers<br><br>- North<br>- Center<br>- South	 <br>4 (26.7)<br>5 (33.3)<br>6 (40.0)

How many patients diagnosed with Crohn's disease with small bowel involvement (L1, L3 or L4 according to Montreal classification) are being treated at your center?<br><br>- Less than 50<br>- Between 50 and 200<br>- Between 200 and 400<br>- More than 400	8 (17.0)<br>16 (34.0)<br>4 (8.5)<br>19 (40.4)
How many of these patients had one or more intestinal resection surgeries?<br><br>- Between 0 and 5%<br>- Between 5 and 15%<br>- Between 15 and 30%<br>- More than 30%	6 (12.8)<br>17 (36.2)<br>13 (27.7)<br>11 (23.4)

Table S1 – Characteristics of the survey respondents – English translation

***indirect data**

Question	N=47	Academic<br><br>N=23	Non-academic<br>N=24	P	Ted prescribers<br>N=15	Ted non prescribers<br>N=32	P	>400 CD with SB involvement<br>N=19	<400 CD with SB involvement<br>N=28	P
**Di quanti di questi pazienti (CD L1, L3, L4 operati) è disponibile un dato di misurazione diretta (chirurgica) dell'intestino residuo?**<br>- 0 – 25%<br>- 25 – 50%<br>- 50 – 75%<br>- 75 – 100%	20 (42.6)<br>12 (25.5)<br>10 (21.3)<br>5 (10.6)	6 (26.1)<br>7 (30.4)<br>7 (30.4)<br>3 (13.0)	14 (58.3)<br>5 (20.8)<br>3 (12.5)<br>2 (8.3)	0.088*	5 (33.3)<br>5 (33.3)<br>4 (26.7)<br>1 (6.7)	15 (46.9)<br>7 (21.9)<br>6 (18.8)<br>4 (12.5)	1.000*	8 (42.1)<br>4 (21.0)<br>5 (26.3)<br>2 (10.5)	12 (42.9)<br>8 (28.6)<br>5 (17.9)<br>3 (10.7)	0,751<br>*
**Di quanti di questi pazienti (operati) è disponibile un dato di misurazione diretta (radiologica) dell'intestino residuo?**<br>- 0 – 25%<br>- 25 – 50%<br>- 50 – 75%<br>- 75 – 100%	25 (53.2)<br>9 (19.1)<br>9 (19.1)<br>4 (8.5)	15 (65.2)<br>4 (17.4)<br>3 (13.0)<br>2 (8.7)	10 (40.0)<br>5 (20.8)<br>6 (25.0)<br>2 (8.3)	0.290*	10 (66.7)<br>1 (6.7)<br>4 (26.7)<br>0 (0)	15 (46.9)<br>8 (25.0)<br>5 (15.6)<br>4 (12.5)	1.000*	10 (52.6)<br>4 (21.0)<br>3 (15.8)<br>2 (10.5)	15 (53.6)<br>5 (17.9)<br>6 (21.4)<br>2 (7.1)	1.000<br>*
**Di quanti di questi pazienti (operati) è disponibile un dato indiretto (citrullinemia, test di assorbimento con D-xilosio) della superficie assorbente dell'intestino residuo?**<br>- 0 – 25%<br>- 25 – 50%<br>- 50 – 75%<br>- 75 – 100%	45 (95.7)<br>2 (4.3)<br>0 (0)<br>0 (0)	-	-	NA	-	-	NA	-	-	NA
**Quanti pazienti con diagnosi di malattia di Crohn con coinvolgimento dell'intestino tenue (L1, L3 o L4 sec classificazione di Montreal) hanno un'insufficienza intestinale (definita dalla necessità di supporto nutrizionale parenterale**										

**per garantire il mantenimento di indici nutrizionali nei limiti)?indicare %**	1.0% (0.0%-2.0%)**	2.0% (1.0%-5.0%)	0.13% (0.0%-1.0%)	0.02	2.0% (1.0%-2.0%)**	0.5% (0.0%-2.0%)	0.12	0.5% (0.0%-2.0%)**	1.5% (0.75%-5%)**	0.056
**Dei pazienti con malattia di Crohn con insufficienza intestinale, quanti hanno necessità di nutrizione parenterale per più di 3 giorni a settimana?**<br>- 0 – 25%<br>- 25 – 50%<br>- 50 – 75%<br>- 75 – 100%	30 (63.9)<br>8 (17.0)<br>9 (19.1)<br>0 (0)	20 (87.0)<br>3 (13.0)<br>1 (4.3)<br>0 (0)	10 (41.7)<br>5 (20.8)<br>8 (33.3)<br>0 (0)	0.724*	7 (46.7)<br>5 (33.3)<br>3 (20.0)<br>0 (0)	23 (71.9)<br>3 (9.4)<br>6 (18.8)<br>0 (0)	1.000*	10 (52.6)<br>3 (15.8)<br>6 (31.6)<br>0 (0)	20 (71.4)<br>5 (17.9)<br>3 (10.7)<br>0 (0)	0.016 *
**Di questi pazienti, quanti sono avviati ad un follow-up multidisciplinare che coinvolge anche un nutrizionista?**<br>- 0 – 25%<br>- 25 – 50%<br>- 50 – 75%<br>- 75 – 100%	20 (42.6)<br>2 (4.3)<br>3 (6.4)<br>22 (46.9)	4 (17.4)<br>2 (8.7)<br>3 (13.0)<br>14 (60.9)	16 (66.7)<br>0 (0)<br>0 (0)<br>8 (33.3)	0.008*	5 (33.3)<br>1 (6.7)<br>0 (0)<br>9 (60.0)	15 (46.9)<br>1 (3.1)<br>3 (9.4)<br>13 (40.6)	0.550*	6 (31.6)<br>0 (0)<br>1 (5.3)<br>12 (63.2)	14 (50.0)<br>2 (7.1)<br>2 (7.1)<br>10 (35.7)	0.136 *
**Quanti pazienti con malattia di Crohn in cura presso il Suo Centro hanno una diagnosi di sindrome dell'intestino corto?**<br>- <10<br>- 10-30<br>- 30-50<br>- 50-100	37 (78.7)<br>8 (17.0)<br>1 (2.1)<br>1 (2.1)	17 (73.9)<br>4 (17.4)<br>1 (4.3)<br>1 (4.3)	20 (83.3)<br>4 (16.7)<br>0 (0)<br>0 (0)	0.494***	12 (80.0)<br>2 (13.3)<br>1 (6.7)<br>0 (0)	25 (78.1)<br>6 (18.8)<br>0 (0)<br>1 (3.1)	1.000***	14 (73.7)<br>3 (15.8)<br>1 (5.3)<br>1 (5.3)	23 (82.1)<br>5 (17.6)<br>0 (0)<br>0 (0)	0.496 ***
**Quanti pazienti con diagnosi di sindrome dell'intestino corto e malattia di Crohn hanno una digiunostomia terminale (sindrome dell'intestino corto di tipo 1)?**<br>- 0 – 25%<br>- 25 – 50%<br>- 50 – 75%<br>- 75 – 100%	42 (89.4)<br>3 (6.4)<br>1 (2.1)<br>1 (2.1)	18 (78.3)<br>3 (13.0)<br>1 (4.3)<br>1 (4.3)	24 (100)<br>0 (0)<br>0 (0)<br>0 (0)	NA	12 (80.0)<br>1 (6.7)<br>1 (6.7)<br>1 (6.7)	30 (93.4)<br>2 (6.3)<br>0 (0)<br>0 (0)	NA	16 (84.2)<br>2 (10.5)<br>0 (0)<br>1 (5.3)	26 (92.9)<br>1 (3.6)<br>1 (3.6)<br>0 (0)	NA
**Quali metodi vengono generalmente utilizzati nella valutazione dello status nutrizionale dei pazienti?**<br>- CB<br>- CS<br>- CBSR<br>- C	29 (61.7)<br>2 (4.3)<br>13 (27.7)<br>3 (6.4)	9 (39.1)<br>2 (8.7)<br>11 (47.8)<br>1 (4.3)	20 (83.3)<br>0 (0)<br>2 (8.3)<br>2 (8.3)	NA	5 (33.3)<br>1 (6.7)<br>9 (60.0)<br>0 (0)	24 (75.0)<br>1 (3.1)<br>4 (12.5)<br>3 (9.4)	NA	9 (47.4)<br>1 (5.3)<br>8 (42.1)<br>1 (5.3)	20 (71.4)<br>1 (3.6)<br>5 (17.9)<br>2 (7.1)	NA
**Quanti pazienti con malattia di Crohn e sindrome dell'intestino corto hanno manifestato eventi avversi correlati alla nutrizione parenterale (es: complicanze catetere-relate, intestinal failure-associated liver disease)? Indicare % o numero assoluto**	NA	NA	NA	NA	NA	NA	NA	NA	NA	NA
**Quanti pazienti con malattia di Crohn e sindrome dell'intestino corto assumono terapia sintomatica anti-secretoria (es: PPI, octreotide, budesonide), antiperistaltica (es: loperamide, codeina, clonidina) o per il malassorbimento di specifici nutrienti (es: pancrelipasi, lattasi)?**<br>- 0 – 25%<br>- 25 – 50%<br>- 50 – 75%<br>- 75 – 100%	N=42<br><br>19 (40.4)<br>10 (21.3)<br>6 (12.8)	N=22<br><br>6 (27.3)<br>8 (36.4)<br>2 (9.1)	N=20<br><br>13 (65.0)<br>2 (10.0)<br>4 (20.0)	0.514*	N=13<br><br>2 (15.4)<br>3 (23.1)<br>4 (30.8)	N=29<br><br>17 (58.6)<br>7 (24.1)<br>2 (6.9)	0.009*	N=17<br><br>6 (35.3)<br>5 (29.4)<br>1 (5.9)	N=25<br><br>13 (52.0)<br>5 (20.0)<br>5 (20.0)	0.738 *

	7 (14.9)	6 (27.3)	1 (5.0)		4 (30.8)	3 (10.3)		5 (29.4)	2 (8.0)	

Table S2 – questions related to epidemiology and clinical management and relative subgroup analysis – original version

*Calcolato con test Esatto di Fisher suddividendo in 2 gruppi (> 50% e <50%)
**Median (interquartile range)
*** Calcolato con test Esatto di Fisher suddividendo in 2 gruppi (<10 SBS e >10 SBS)

NA= non applicabile; C= clinico; CB= clinico e biochimico (albumina, B12, folato, profilo marziale, transferrina, transtiretina, prealbumina, citrullina); CS= clinico e strumentale (es. analisi bioimpedenziometrica); CBSR= criteri clinici, biochimici, strumentali e radiologici (misurazione del muscolo psoas o dei muscoli paravertebrali).

Question	N=47	Academic<br>N=23	Non-academic<br>N=24	P	Ted prescribers<br>N=15	Ted non prescribers<br>N=32	P	>400 CD with SB involvement<br>N=19	<400 CD with SB involvement<br>N=28	P
**Of how many of these patients (CD L1, L3, L4 operated) is direct (surgical) measurement data of residual bowel available?**<br>- 0 – 25%<br>- 25 – 50%<br>- 50 – 75%<br>- 75 – 100%	20 (42.6)<br>12 (25.5)<br>10 (21.3)<br>5 (10.6)	6 (26.1)<br>7 (30.4)<br>7 (30.4)<br>3 (13.0)	14 (58.3)<br>5 (20.8)<br>3 (12.5)<br>2 (8.3)	0.088*	5 (33.3)<br>5 (33.3)<br>4 (26.7)<br>1 (6.7)	15 (46.9)<br>7 (21.9)<br>6 (18.8)<br>4 (12.5)	1,00<br>*	8 (42.1)<br>4 (21.0)<br>5 (26.3)<br>2 (10.5)	12 (42.9)<br>8 (28.6)<br>5 (17.9)<br>3 (10.7)	0,751<br>*
**Of how many of these patients (CD L1, L3, L4 operated) is direct (radiological) measurement data of residual bowel available?**<br>- 0 – 25%<br>- 25 – 50%<br>- 50 – 75%<br>- 75 – 100%	25 (53.2)<br>9 (19.1)<br>9 (19.1)<br>4 (8.5)	15 (65.2)<br>4 (17.4)<br>3 (13.0)<br>2 (8.7)	10 (40.0)<br>5 (20.8)<br>6 (25.0)<br>2 (8.3)	0.290*	10 (66.7)<br>1 (6.7)<br>4 (26.7)<br>0 (0)	15 (46.9)<br>8 (25.0)<br>5 (15.6)<br>4 (12.5)	1,00<br>*	10 (52.6)<br>4 (21.0)<br>3 (15.8)<br>2 (10.5)	15 (53.6)<br>5 (17.9)<br>6 (21.4)<br>2 (7.1)	1,000<br>*
**Of how many of these patients (CD L1, L3, L4 operated) is indirect data (citrullinemia, D-xylose absorption test) of the absorptive surface area of the residual intestine available?**<br>- 0 – 25%<br>- 25 – 50%<br>- 50 – 75%<br>- 75 – 100%	45 (95.7)<br>2 (4.3)<br>0 (0)<br>0 (0)	-	-	NA	-	-	NA	-	-	NA
**How many patients diagnosed with Crohn's disease with small bowel involvement (L1, L3 or L4 according to Montreal classification) have intestinal insufficiency (defined by the need for parenteral nutritional support to ensure maintenance of nutritional indices within limits)? give %**	1.0% (0.0%-2.0%)**	2.0% (1.0%-5.0%)	0.13% (0.0%-1.0%)	0.02	2.0% (1.0%-2.0%)**	0.5% (0.0%-2.0%)	0.12	0.5% (0.0%-2.0%)**	1.5% (0.75%-5%)**	0.056
**Of Crohn's disease patients with intestinal failure, how many require parenteral nutrition for more than 3 days per week?**										

- 0 – 25%<br>- 25 – 50%<br>- 50 – 75%<br>- 75 – 100%	30 (63.9)<br>8 (17.0)<br>9 (19.1)<br>0 (0)	20 (87.0)<br>3 (13.0)<br>1 (4.3)<br>0 (0)	10 (41.7)<br>5 (20.8)<br>8 (33.3)<br>0 (0)	0.724*	7 (46.7)<br>5 (33.3)<br>3 (20.0)<br>0 (0)	23 (71.9)<br>3 (9.4)<br>6 (18.8)<br>0 (0)	1.00<br>*	10 (52.6)<br>3 (15.8)<br>6 (31.6)<br>0 (0)	20 (71.4)<br>5 (17.9)<br>3 (10.7)<br>0 (0)	0.016<br>*
**Of these patients, how many are initiated to multidisciplinary follow-up involving a nutritionist?**<br>- 0 – 25%<br>- 25 – 50%<br>- 50 – 75%<br>- 75 – 100%	20 (42.6)<br>2 (4.3)<br>3 (6.4)<br>22 (46.9)	4 (17.4)<br>2 (8.7)<br>3 (13.0)<br>14 (60.9)	16 (66.7)<br>0 (0)<br>0 (0)<br>8 (33.3)	0.008*	5 (33.3)<br>1 (6.7)<br>0 (0)<br>9 (60.0)	15 (46.9)<br>1 (3.1)<br>3 (9.4)<br>13 (40.6)	0.55<br>*	6 (31.6)<br>0 (0)<br>1 (5.3)<br>12 (63.2)	14 (50.0)<br>2 (7.1)<br>2 (7.1)<br>10 (35.7)	0.136<br>*
**How many Crohn's disease patients treated at your center have a diagnosis of short bowel syndrome?**<br>- <10<br>- 10-30<br>- 30-50<br>- 50-100	37 (78.7)<br>8 (17.0)<br>1 (2.1)<br>1 (2.1)	17 (73.9)<br>4 (17.4)<br>1 (4.3)<br>1 (4.3)	20 (83.3)<br>4 (16.7)<br>0 (0)<br>0 (0)	0.494*<br>**	12 (80.0)<br>2 (13.3)<br>1 (6.7)<br>0 (0)	25 (78.1)<br>6 (18.8)<br>0 (0)<br>1 (3.1)	1.00<br>***	14 (73.7)<br>3 (15.8)<br>1 (5.3)<br>1 (5.3)	23 (82.1)<br>5 (17.6)<br>0 (0)<br>0 (0)	0.496<br>***
**How many patients diagnosed with short bowel syndrome and Crohn's disease have terminal jejunostomy (short bowel syndrome type 1)?**<br>- 0 – 25%<br>- 25 – 50%<br>- 50 – 75%<br>- 75 – 100%	42 (89.4)<br>3 (6.4)<br>1 (2.1)<br>1 (2.1)	18 (78.3)<br>3 (13.0)<br>1 (4.3)<br>1 (4.3)	24 (100)<br>0 (0)<br>0 (0)<br>0 (0)	NA	12 (80.0)<br>1 (6.7)<br>1 (6.7)<br>1 (6.7)	30 (93.4)<br>2 (6.3)<br>0 (0)<br>0 (0)	NA	16 (84.2)<br>2 (10.5)<br>0 (0)<br>1 (5.3)	26 (92.9)<br>1 (3.6)<br>1 (3.6)<br>0 (0)	NA
**What methods are generally used in assessing the nutritional status of patients?**<br>- CB<br>- CS<br>- CBSR<br>- C	29 (61.7)<br>2 (4.3)<br>13 (27.7)<br>3 (6.4)	9 (39.1)<br>2 (8.7)<br>11 (47.8)<br>1 (4.3)	20 (83.3)<br>0 (0)<br>2 (8.3)<br>2 (8.3)	NA	5 (33.3)<br>1 (6.7)<br>9 (60.0)<br>0 (0)	24 (75.0)<br>1 (3.1)<br>4 (12.5)<br>3 (9.4)	NA	9 (47.4)<br>1 (5.3)<br>8 (42.1)<br>1 (5.3)	20 (71.4)<br>1 (3.6)<br>5 (17.9)<br>2 (7.1)	NA
**How many patients with Crohn's disease and short bowel syndrome have experienced adverse events related to parenteral nutrition (e.g. catheter-related complications, intestinal failure-associated liver disease)? Indicate % or absolute number**	NA	NA	NA	NA	NA	NA	NA	NA	NA	NA
**How many patients with Crohn's disease and short bowel syndrome take symptomatic anti-secretory therapy (e.g. PPI, octreotide, budesonide), anti-peristaltic therapy (e.g. loperamide, codeine, clonidine) or for malabsorption of specific nutrients (e.g. pancrelipase, lactase)?**<br>- 0 – 25%<br>- 25 – 50%<br>- 50 – 75%<br>- 75 – 100%	N=42<br>19 (40.4)<br>10 (21.3)<br>6 (12.8)<br>7 (14.9)	N=22<br>6 (27.3)<br>8 (36.4)<br>2 (9.1)<br>6 (27.3)	N=20<br>13 (65.0)<br>2 (10.0)<br>4 (20.0)<br>1 (5.0)	0.514*	N=13<br>2 (15.4)<br>3 (23.1)<br>4 (30.8)<br>4 (30.8)	N=29<br>17 (58.6)<br>7 (24.1)<br>2 (6.9)<br>3 (10.3)	0.00<br>9*	N=17<br>6 (35.3)<br>5 (29.4)<br>1 (5.9)<br>5 (29.4)	N=25<br>13 (52.0)<br>5 (20.0)<br>5 (20.0)<br>2 (8.0)	0.738<br>*

Table S2 – questions related to epidemiology and clinical management and relative subgroup analysis – English version

*Calculated with Fisher's Exact test by dividing into 2 groups (>50% and <50%)

**Median (interquartile range).

***Calculated with Fisher's Exact test by dividing into 2 groups (<10 SBS and >10 SBS)

NA=not applicable; C= clinical; CB= clinical and biochemical (Albumin, B12, folate, martial profile, transferrin, transthyretin, prealbumin, citrulline); CS= clinical and instrumental (e.g. bioimpedance analysis); CBSR= Clinical, biochemical, instrumental, and radiological criteria (measurement of psoas muscle or paravertebral muscles).

Domanda	N = rispondenti alla singola domanda
**Quanti pazienti con diagnosi di sindrome dell'intestino corto con malattia di Crohn in cura presso il Suo centro sono in terapia con teduglutide?**	0.0% (0.0%-0.5%)*
	N=11
**Quanto è la durata massima della terapia con teduglutide dei pazienti seguiti presso il Suo Centro?**	
- <6 mesi	0 (0)
- 6-12 mesi	4 (36.4)
- 12-24 mesi	3 (27.3)
- >24 mesi	4 (36.4)
	N=11
**Quanti di questi pazienti hanno raggiunto l'endpoint terapeutico (riduzione di almeno il 30% della necessità di nutrizione parenterale settimanale)?**	
- 0 – 25%	2 ()
- 25 – 50%	0 (0)
- 50 – 75%	6 ()
- 75 – 100%	3 ()
	N=10
**Riguardo al ruolo anti-infiammatorio attribuito alla teduglutide, in quanti pazienti si è assistito ad una riduzione del Crohn's Disease Activity Index (CDAI), dall'introduzione di teduglutide?**	
- 0 – 25%	4 (40.0)
- 25 – 50%	3 (30.0)
- 50 – 75%	2 (20.0)
- 75 – 100%	1 (10.0)
	N=26
**In quanti casi è stata esclusa teduglutide come potenziale terapia in pazienti con malattia di Crohn attiva a causa del teorico rischio che le sue azioni intestinotrofiche possano esacerbare l'infiammazione ed il rischio di cancro?**	
- 0 – 25%	20 (76.9)
- 25 – 50%	3 (11.5)
- 50 – 75%	2 (7.7)
- 75 – 100%	1 (3.8)
**Quanti pazienti hanno dovuto sospendere la terapia con teduglutide per l'insorgenza di eventi avversi (es: addominalgia severa, distensione addominale, nausea, vomito) indicare % o numero assoluto**	NA

Table S3 – Questions related to teduglutide therapy – original version

*Median (interquartile range)

Question	N = respondents to the single questions
**How many patients diagnosed with short bowel syndrome with Crohn's disease being treated at your center are on teduglutide therapy?**	0.0% (0.0%-0.5%)*
	N=11
**How long is the maximum duration of teduglutide therapy of patients followed at your center?**	
- **<6 months**	0 (0)
- **6-12 months**	4 (36.4)
- **12-24 months**	3 (27.3)
- **>24 months**	4 (36.4)
	N=11
**How many of these patients achieved the therapeutic endpoint (at least 30% reduction in the need for weekly parenteral nutrition)?**	
- **0 - 25%**	2 ()
- **25 - 50%**	0 (0)
- **50 - 75%**	6 ()
- **75 - 100%**	3 ()
	N=10
**Regarding the anti-inflammatory role attributed to teduglutide, in how many patients has there been a reduction in Crohn's Disease Activity Index (CDAI), since the introduction of teduglutide?**	
- 0 – 25%	4 (40.0)
- 25 – 50%	3 (30.0)
- 50 – 75%	2 (20.0)
- 75 – 100%	1 (10.0)
**In how many cases has teduglutide been excluded as a potential therapy in patients with active Crohn's disease because of the theoretical risk that its intestinotrophic actions may exacerbate inflammation and cancer risk?**	N=26

- 0 – 25%<br>- 25 – 50%<br>- 50 – 75%<br>- 75 – 100%	20 (76.9)<br>3 (11.5)<br>2 (7.7)<br>1 (3.8)
**How many patients had to discontinue teduglutide therapy due to the occurrence of adverse events (e.g., severe abdominalgia, abdominal distension, nausea, vomiting) indicate % or absolute number**	NA

Table S3 – Questions related to teduglutide therapy – English version

*Median (interquartile range)

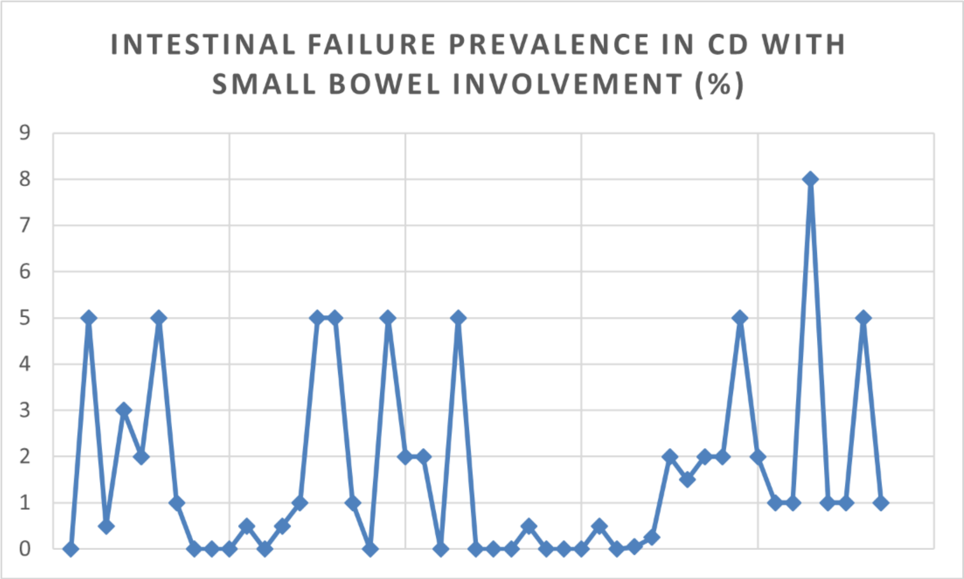

Figure S1 – The estimated prevalence (% on the y-axis) of intestinal failure in Crohn's Disease (CD) with small bowel involvement, as reported by each of the 47 responding centers (x-axis).
